# Immunohistochemical study of N-epsilon-carboxymethyl lysine (CML) in human brain: relation to vascular dementia

**DOI:** 10.1186/1471-2377-7-35

**Published:** 2007-10-16

**Authors:** Louise Southern, Jonathan Williams, Margaret M Esiri

**Affiliations:** 1 Department of Clinical Neurology, University of Oxford, West Wing, John Radcliffe Hospital, Oxford UK; 2 Department of Pharmacology, University of Oxford, Oxford, UK; 3 Department of Neuropathology, Oxford Radcliffe NHS Trust, John Radcliffe Hospital, Oxford, UK

## Abstract

**Background:**

Advanced glycation end-products (AGEs) and their receptor (RAGE) occur in dementia of the Alzheimer's type and diabetic microvascular disease. Accumulation of AGEs relates to risk factors for vascular dementia with ageing, including hypertension and diabetes. Cognitive dysfunction in vascular dementia may relate to microvascular disease resembling that in diabetes. We tested if, among people with cerebrovascular disease, (1) those with dementia have higher levels of neuronal and vascular AGEs and (2) if cognitive dysfunction depends on neuronal and/or vascular AGE levels.

**Methods:**

Brain Sections from 25 cases of the OPTIMA (Oxford Project to Investigate Memory and Ageing) cohort, with varying degrees of cerebrovascular pathology and cognitive dysfunction (but only minimal Alzheimer type pathology) were immunostained for N^*ε*^-(carboxymethyl)-lysine (CML), the most abundant AGE. The level of staining in vessels and neurons in the cortex, white matter and basal ganglia was compared to neuropsychological and other clinical measures.

**Results:**

The probability of cortical neurons staining positive for CML was higher in cases with worse cognition (p = 0.01) or a history of hypertension (p = 0.028). Additionally, vascular CML staining related to cognitive impairment (p = 0.02) and a history of diabetes (p = 0.007). Neuronal CML staining in the basal ganglia related to a history of hypertension (p = 0.002).

**Conclusion:**

CML staining in cortical neurons and cerebral vessels is related to the severity of cognitive impairment in people with cerebrovascular disease and only minimal Alzheimer pathology. These findings support the possibility that cerebral accumulation of AGEs may contribute to dementia in people with cerebrovascular disease.

## Background

Advanced glycation end-products (AGEs) may contribute to the aetiology of many disease processes including Alzheimer's disease (AD) [1-9] and the vascular complications of diabetes [10]. The microvascular changes in diabetes bear some similarity to the microvascular disease observed in patients with vascular dementia (VD) [11]. VD shares many risk factors with AD such as age, hypertension, diabetes mellitus and hyperhomocysteinemia [12]. Hence, VD often co-occurs with Alzheimer's disease (AD). Previous work has shown that even in cases where AD pathology is only mild, VD may promote cognitive impairment [13]. Hence, if AGEs contribute to microvascular damage, then they may also relate to cognitive impairment. The present study tested this hypothesis.

AGEs are adducts or crosslinks which can form non-enzymatically between reducing sugars such as glucose, and other moieties such as lipids, nucleic acids or proteins. The classical pathway of AGE formation, the Maillard (browning) reaction involves a glucose-protein condensation reaction to form Schiff base adducts, which are subject to Amadori rearrangement. Some Amadori products convert to AGEs, which are characteristically fluorescent, pigmented adducts that can cross link with proteins and persist for the lifetime of the modified substrate. Examples include N^*ε*^-(carboxymethyl)-lysine (CML), pentosidine, pyrraline and crosslines A and B, all of which have been identified in brain using immunohistochemical methods [5,8,14].

The degree of AGE accumulation in a tissue depends on the rates of AGE formation and degradation. Hyperglycemia and oxidative stress accelerate the former, while the latter depends on the rates of protein turnover, ligation to macrophage scavenger receptors (MSR) [15] and renal clearance. Probably, the accumulation of AGEs during natural ageing [16] is due to the time-dependent nature of advanced glycation coupled with increased oxidative stress and a progressive reduction in the capacity to neutralise oxidative stress. AGEs have diverse detrimental effects. They can damage proteins directly and enhance oxidative stress via specific receptors (RAGE).

CML is the most abundant AGE *in vivo *[14]. It accumulates in vascular tissue and atherosclerotic and diabetic lesions [17,18]. Levels of CML in serum are higher in diabetics with retinopathy and microalbuminaemia than in those without these complications [19,20]. The amount of CML detectable in cortical neurons increases with ageing [4] and even more so in AD [1-9]. This increased accumulation of AGEs in the brains of AD patients [5,8,14] may index neuronal damage due to glycation and oxidative stress.

Our present aim was to test if CML expression in the brain in elderly subjects with cerebrovascular disease relates to cognitive dysfunction. Accordingly, we analysed immunocytochemical expression of CML in cerebral neurons and blood vessel walls in autopsy samples from people with cerebrovascular disease with or without cognitive dysfunction and explored how CML expression related to presence of risk factors for vascular dementia. Since vascular and Alzheimer pathology often co-exist [13], to clarify the relation of cognitive impairment to AGEs requires careful control of its relation to Alzheimer pathology. To this end, we studied only samples from individuals with minimal Alzheimer pathology and we covaried the severity of that pathology. Finally, to test whether cognitive impairment relates specifically to vascular AGE staining, we studied CML staining not only in vessel walls in subcortical white matter and basal ganglia but also in neurons.

## Methods

### Subjects

We examined formalin-fixed, paraffin-embedded sections from post-mortem samples of the frontal cortex (Brodmann area 46), and white matter and basal ganglia from 25 cases (13 male) in the Oxford Project to Investigate Memory and Ageing (OPTIMA). This is a longitudinal study of demented and non-demented people, involving over 900 subjects recruited over 18 years who underwent detailed annual clinical neuropsychological assessments [21]. The latter included use of CAMCOG [22] and MMSE. Autopsy was consented to in 94% of the first 250 subjects to die. The age at death ranged from 64–92 years (mean 78.9 years). All participants gave informed consent at entry to the study and all autopsies were performed with consent from the next of kin. This study had the approval of a National Health Service local Research Ethics Committee.

Brain sections from the first 250 cases to die with autopsy consent in the OPTIMA study were assessed semi-quantitatively for Alzheimer and vascular pathology. Assessment of Alzheimer pathology followed the CERAD protocol [23] and Braak staging [24]. Vascular pathology was assessed as described by Esiri *et al *1997 [11]. Presence or absence of Lewy bodies in substantia nigra and cerebral cortex was also recorded. The 25 cases studied (see additional file 1) were selected on the pathological basis that they were the first cases to die that had (1) a Braak stage of Alzheimer pathology no higher than 3 [24] (i.e. subclinical) and their neuritic plaque scores in neocortex were insufficient to meet research criteria for the pathological diagnosis of AD [23]; (2) evidence of cerebrovascular disease with major or minor cerebral infarcts ± subcortical small vessel disease reflected in moderate to severe pallor in myelin-stained sections and/or one or more lacunar infarcts (Table 1 - see additional file 1); (3) no evidence of pathology of any other dementing syndrome.

### Immunocytochemistry for CML

10 μm thick sections were cut from paraffin embedded frontal cortex and white matter (WM) and basal ganglia (BG) blocks and mounted on silane-coated slides. These were dewaxed in Histoclear and rehydrated through graded concentrations of ethanol to water before being treated with a 3% hydrogen peroxide solution in PBS for 30 minutes.

The sections were incubated with 3 drops of proteinase K (DAKO Kit) for 5 minutes. Proteolytic digestion is a well-documented technique used to expose the cross-linked AGE moieties and to enhance AGE immunoreactivity in the human brain [25]. The sections were then incubated with 10% normal horse serum (Vector Laboratories) for 20 minutes to minimise non-specific binding, prior to incubation overnight at 4°C with a 1:500 dilution in tris-phosphate-buffered saline (PBS-T) of anti-CML antibody (NBS Biologicals). This is a mouse monoclonal antibody derived from a splenic lymphocyte cell line from a BALB/c mouse immunised with CML-HAS. It was purified by Protein G affinity chromatography. Sections were washed and incubated with a 1:200 dilution of secondary antibody (mouse IgG, Vector Laboratories) for 30 minutes and Vectastain ABC Kit (Vector Laboratories), a kit to amplify the process, for 45 minutes. Bound antibody was visualised with Immuno Pure Metal Enhanced DAB Substrate Kit, which included 3,3'-diaminobenzidine (DAB) metal concentrate [10x] diluted 1:10 in Stable Peroxide Buffer [1x] (CIB Perbio). After about 4 minutes in DAB solution the products began to appear and the final reaction was then stopped by washing with distilled water before the sections were counterstained with haematoxylin solution.

To confirm specificity, negative control sections were stained as above, replacing the primary antibody with PBS. As expected, these sections did not stain. As a positive control a section of hippocampus was stained (as the CA4 region has repeatedly shown strong staining for CML in elderly subjects in earlier studies [14]. Serial sections (WM and BG) were also stained with Luxol Fast Blue, a myelin stain, and Cresyl Violet to aid anatomical location of white matter and grey matter.

### Microscopic analysis

The outcome measures in this study were the percentage of cortical or basal ganglia neurons immunostained for CML and the semi-quantitative scores for CML immunostaining in vessel walls. These were derived as follows: microscopic analysis was carried out blind to clinical information by one observer (LS). Ten cortical fields at ×400 magnification were viewed and the number of positive and negative nucleolated neurons counted in each field. Counts on two separate occasions of the same fields were within 10% or less of each other for 80% of fields investigated in preliminary analysis. Ten arterioles from white matter and 10 small arteries and arterioles from the sections of basal ganglia were also randomly selected for examination and the staining for CML in their walls categorised as strong (++), weak (+) or absent (-). Each category was expressed as a fraction of the vessels counted. Location of staining in the vessel walls was also recorded.

### Clinical data gathering

Following the completion of microscopic analysis of the slides the following data on each case was obtained from OPTIMA patients' records: age, sex, survival, post mortem delay, history of hypertension defined as a systolic blood pressure above 140 and/or a diastolic blood pressure above 90 [26], diabetes status, CAMCOG score and MMSE score, both at final assessment (Table 1 - see additional file 1), though if there was severe terminal morbidity that meant scores were judged unrepresentative, then the penultimate scores were used in a few subjects. A CAMCOG score below 80 out of a maximum of 107 characterised those with dementia [27]. The clinical dementia diagnostic categories of the cases included in the study were: mixed vascular dementia and AD (n = 8) (two of these cases had a history of stroke); vascular dementia (n = 3); probable AD (n = 1); other dementia not otherwise specified (n = 1); cognitively intact with no other neurological illness (n = 9); cognitively intact with a history of stroke (n = 1).

### Statistical analysis

All analyses used the open source *R *[28] statistical language. Initial analyses of demographic data used Fisher exact and Wilcoxon-Mann-Whitney (WMW) tests. In all analyses, the α-level of significance was *p *< 0.05. We then analysed inter-relations between CML staining and clinical variables in two ways: (a) we modelled dependence of CML staining on clinical variables; (b) we modelled dependence of survival on CML staining.

(a) Analysis of neuronal CML staining used a repeated-measures binomial regression – an extension of the (mixed effects) generalised linear model [29], Generalised linear mixed modelling (GLMM) can account for the correlation of observations (here, the nesting of slides) within individuals. We implemented generalised linear mixed modelling ('lmer') [30] with optimisation using the Laplace approximation. This model analysed the dependence of neuronal CML immunostaining on age, sex, dementia status, MMSE, hypertension and brain region. The lmer model included random effects for individuals and brain region and evaluated the probability of staining in each cell after adjusting for the effects of sex, age, PM delay and other clinical variables. We also tested the relations of the random effects for CML staining with dementia status using Receiver Operating Characteristic (ROC) curves.

Analysis of vessel CML staining used the generalised estimating equations (GEE). This is because the ratings of CML staining were ordinal (absent, weak or strong). GEE can account for the correlation of observations within individuals.

(b) We tested the dependence of survival on CML staining using Weibull regression. These models covaried variables that can influence survival, including gender and dementia status.

## Results

### Clinical characteristics

Of the selected cases, 10 were clinically non-demented at time of death and 15 were demented. The non-demented cases tended, not quite significantly, to be older at death than those with dementia (WMW p = 0.07) (Table 1 - see additional file 1). Five had a history of diabetes, of whom two were demented (Table 1) (OR = 3.3, Fisher exact p = 0.31). Fifteen cases had a history of hypertension, of whom eight had dementia (OR = 1.95, Fisher exact p = 0.68). Three cases had clinical evidence of a stroke (one non-demented, two demented).

The cause of death tended, not quite significantly, to be pneumonia more often for cases with dementia than those who were non-demented (OR = 6.0, Fisher exact p = 0.07). Mean post mortem delay did not differ between demented and non-demented cases (mean = 61 hrs, range 23–144) (WMW p = 0.31). Overall vascular pathology (the sum of different types of vascular pathology) did not differ with dementia status (WMW p = 0.3), history of diabetes (WMW p = 0.68) or hypertension (WMW p = 0.31). Nor did individual type of vascular pathology relate specifically to ante mortem clinical variables (all Fisher exact *p*s > 0.2) (Table 1). Cases with a history of diabetes were younger than those without (WMW p = 0.005 for diabetes) and there was a similar trend for history of hypertension (WMW p = 0.08) (see Table 1).

### Cortical neuronal CML immunostaining: CAMCOG scores, hypertension and survival

**Figure 1:**
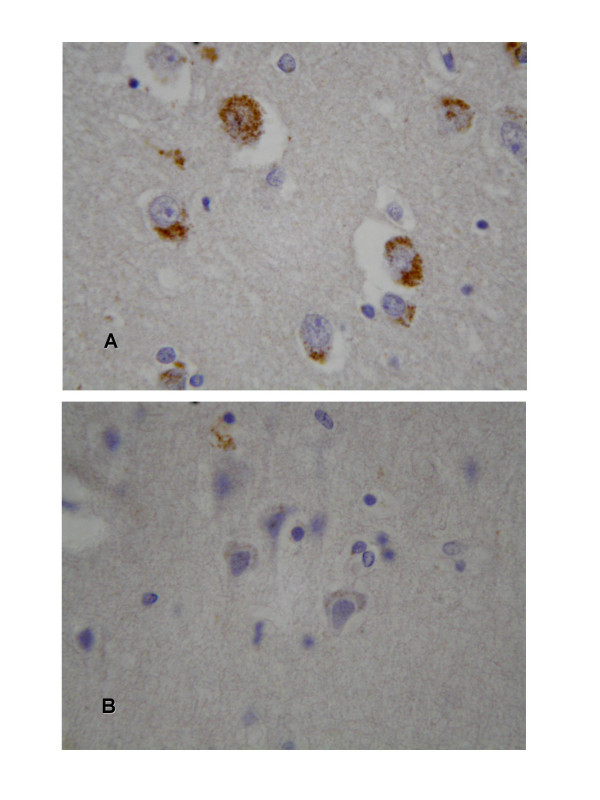
Examples of positively (a) and negatively (b) immunostained cortical neurons for CML. Counterstained with haematoxylin.

All cases showed at least some degree of neuronal staining in both basal ganglia and cortex. (In 2 cases cortical sections were excluded from analysis because of technical artefacts.) There was evidence of lipofuscin granules in many neurons in Nissl-stained sections and these were heavily stained suggesting they are a site of CML accumulation. This agrees with earlier reports on CML immunostaining in neurons [2,5,9]. Large neurons in cortex and basal ganglia were those most commonly identified as positively stained for CML (Figure 1).

**Figure 2:**
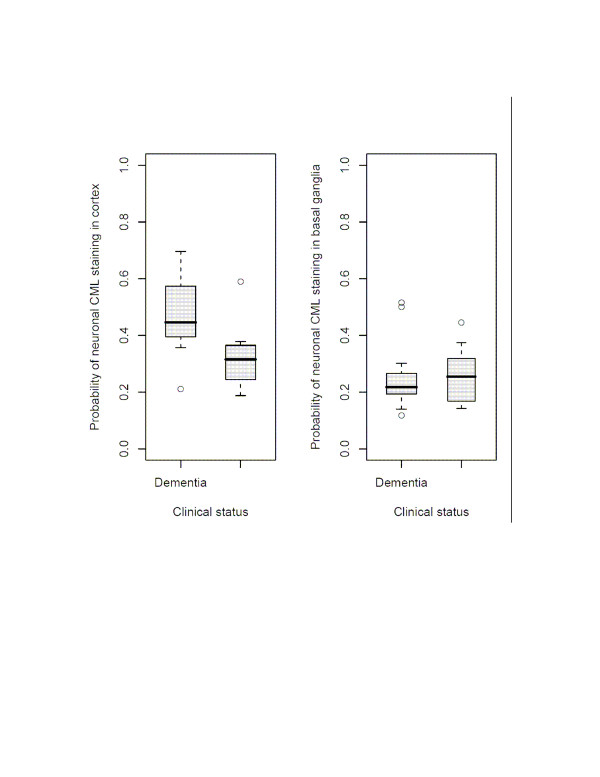
(a) (left) Probability of cortical neuronal staining vs. clinical dementia status. Box plots compare demented and undemented subjects. (b) (right) Probability of basal ganglia staining vs. clinical dementia status. The probability of CML staining in basal ganglia neurons did not differ between the two groups.

(a) GLMM showed that in the cortex, the proportion of neurones staining positively for CML was higher in cases with dementia (z = 2.56, p = 0.01; Figure 2a). With patient status in the model, cortical neural CML staining also related to history of hypertension (z = 2.20, p = 0.028). These two relationships remained significant if we covaried age at death, gender, history of diabetes, history of hypertension, post mortem delay or Braak stage (none of which were significant). When covarying Braak stage and hypertension, higher probability of CML staining in cortical neurons discriminated patient status (area under ROC curve = 82.6%; for CML staining over 40%, odds ratio of dementia = 21.8, Fisher exact p = 0.0075).

(b) Robust linear modelling indicated that, even after covarying Braak stage and age, Lower CAMCOG scores related to higher probability of CML staining in cortical neurons (t = -2.26, 20df, p = 0.03) and to more CML-stained vessels (t = -2.14, 20df, p = 0.044. (c) Weibull regression found no relation between the probability of cortical neuronal CML staining and survival (z = 1.08, p > 0.25)

### Basal ganglia neuronal CML staining: CAMCOG scores, hypertension and survival

**Figure 3:**
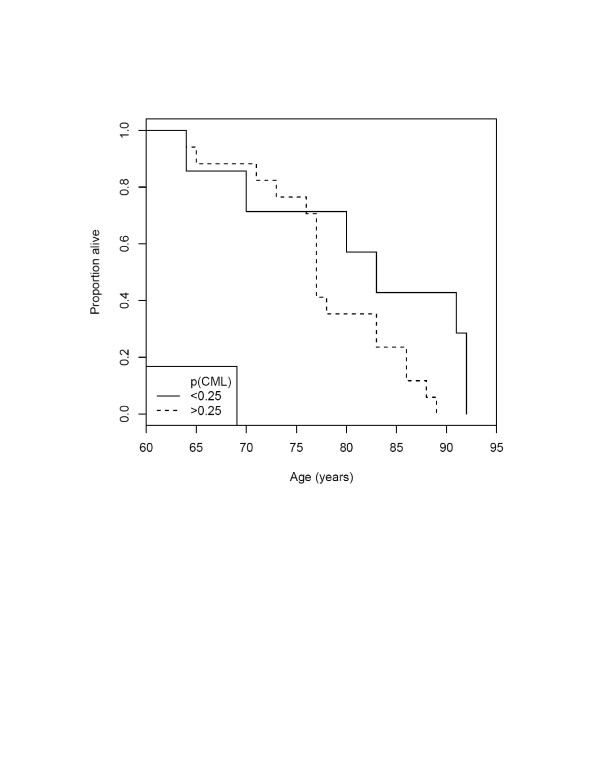
Survival curves for subjects with and without CML immunostaining of basal ganglia neurons in > 25% of neurons.

(a) GLMM indicated that basal ganglia neurones were more likely to stain positively for CML in cases with a history of hypertension (z = 3.09, p = 0.0026; Figure 2b). The relationship of basal ganglia neural CML staining with history of hypertension remained significant if we covaried age at death, gender, history of diabetes or hypertension, post mortem delay or Braak stage (none of which were significant). CML staining in basal ganglia neurons did not discriminate patient status (area under ROC curve ≈50%). (b) Robust linear modeling indicated that CAMCOG scores did not relate to basal ganglia neuronal CML staining (z = -0.41, p = 0.67). (c) Weibull regression showed that higher probability of CML staining in basal ganglia neurones related to shorter survival (z = -2.04, p = 0.041) (Figure 3 illustrates this, dichotomising the probability of CML staining at 0.25). This relationship remained significant (z = -1.97, p = 0.049) when we covaried male gender (z = -2.12, p = 0.034) and patient status (z = -2.43, p = 0.015).

### Vessel CML staining: CAMCOG scores and diabetes

**Figure 4:**
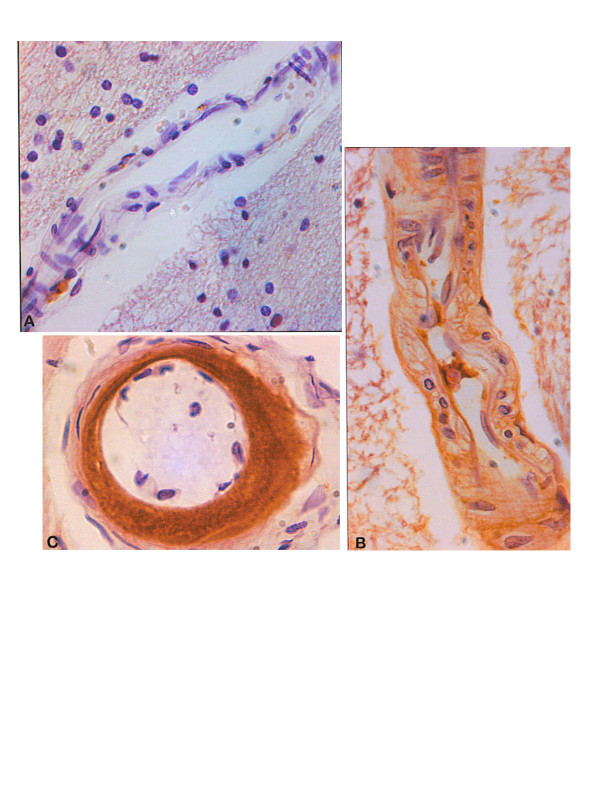
Examples of vessel wall scoring for immunostaining for CML a:++ score; b:+ score; c:-score. Counterstained with haematoxylin.

**Figure 5:**
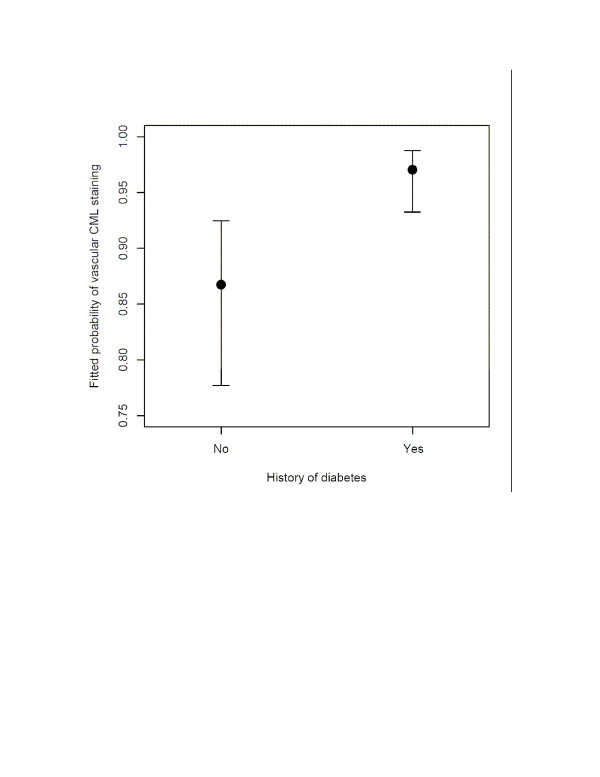
Comparison of the fitted probability (with standard error bars) of vascular CML immunostaining in subjects with and without a history of diabetes.

The staining of the vessels, in both demented and non-demented cases, occurred mainly in the tunica media (Figure 4). (a) GEE analysis showed that the intensity of vascular CML staining related to a history of diabetes (Wald χ^2 ^= 4.00, 1df, p = 0.046) (Figure 5) and inversely to CAMCOG scores (Wald χ^2 ^= 7.1, 1df, p = 0.008) As above, these relations remained significant if we covaried other variables (none of which were significant). (b) Robust linear modeling showed that CAMCOG scores related to both cortical neuronal and vascular CML staining independently in the same model that also covaried Braak stage and age (t = -2.43, 17df, p = 0.026). (c) Weibull regression found no relation between vessel CML staining and survival.

## Discussion

CML expression in microvessels and cortical neurones related to clinical dementia in people in whom cerebrovascular disease was the main pathology. Our study focussed on the relationship between AGE levels and cognitive impairment by controlling concurrent Alzheimer pathology. This control greatly strengthens the support that our results give to the hypothesis that AGEs contribute to cognitive dysfunction in cerebrovascular disease.

One of the main goals of our study was to test the hypothesis of a relationship between expression of RAGE ligands, specifically, CML, in cerebral vessel walls and cognitive dysfunction in pathologically confirmed cerebrovascular disease. The present results support this hypothesis, since vascular CML staining was higher in people with clinical dementia. This relationship was present overall in our sample – all of whom had Braak stages for Alzheimer pathology of 3 or less. However, we also ensured that Alzheimer pathology did not contribute to CML staining, by covarying Braak stage. Even then, the relationship between dementia and vascular CML staining remained significant. Vascular CML staining also related to a history of diabetes, even though there were only 5 cases with diabetes in our study. This finding is consistent with previous observations that AGE/RAGE expression in small blood vessel walls and in blood correlates positively with end organ damage in clinical and experimental diabetes [31-35,19,20].

The other main goal of our study was to see if neuronal CML staining was related to cognitive impairment in cerebrovascular disease. We found that CML staining of cortical neurones related both to dementia and hypertension. The parallel relations of both vascular and cortical neuronal CML staining with dementia raise the possibility that microvascular pathology due to AGEs may be a proxy for their direct effects in causing cortical damage; and it is this damage which results in cognitive dysfunction. CML staining in basal ganglia neurons did not relate to dementia, but related more strongly to a history of hypertension. This is consistent with previous findings that AGE accumulation is greater in hypertension [36-39].

Even though neuronal CML staining in the basal ganglia did not relate to cognitive impairment, it did relate to survival. This raises the possibility that CML staining here may be functionally important. Possibly, some symptoms in the elderly that relate to basal ganglia dysfunction but are not caused by Parkinson's disease pathology may be due to AGE accumulation in basal ganglia neurons. A separate study would be needed to explore this possibility.

The fact that our study was small and cross-sectional makes the direction of the relationship between dementia and cortical immunostaining for CML uncertain. Our statistical analyses are consistent with the possibility that CML accumulation could, in principle, be a cause or a consequence of vascular dementia. In favour of a causative role is the ability of CML to bind to its receptor RAGE and trigger cellular activation and oxidant stress. RAGE has many other ligands and CML can bind to other receptors so, although RAGE and its ligands tend to be detected together [40] the relationship between CML expression and cellular consequences of RAGE activation may not be straightforward. CML and related AGEs may also be able to damage neurons because they produce alterations in affected proteins that can lead to enzyme inactivation and protein denaturation directly [41]. Glycation of proteins also interferes with their degradation [42]. Furthermore, damaging reactive oxygen species are a by-product of glycation [8,17,43,44]. It may also result from lipid peroxidation [45]. Therefore an alternative interpretation to CML being viewed as potentially causative in vascular dementia is that it accumulates as a consequence of ischemia/hypoxia and the accompanying oxidative stress experienced by neurons as a result of cerebrovascular disease [17].

If AGEs contribute to causing cognitive impairment then drugs that prevent or repair protein damage caused by glycation may provide treatment or prevention of VD [41]. Irrespective of whether enhanced CML expression in cortical neurons is a cause or consequence of vascular dementia there is a possibility that further analysis of AGEs may assist in its diagnosis. Cerebrospinal fluid profiles of glycation adduct residues and free adducts can discriminate cases of AD from age-matched healthy subjects [46]. A recent study has described reduced levels of soluble RAGE in plasma both in AD and vascular dementia [47]. Further studies should test if AGE-related moieties can differentiate AD and vascular dementia.

## Competing interests

The author(s) declare that they have no competing interests.

## Authors' contributions

LS contributed to the study design, collected the microscopic data, assisted with collecting the clinical data and scrutinised and suggested modifications to the manuscript.

JW contributed to the study design, led the collection of clinical data, performed the statistical analyses and scrutinised and modified the manuscript.

MME suggested the study, contributed to its design and prepared a first draft of the manuscript.

All authors have read and approved the final manuscript.

## Pre-publication history

The pre-publication history for this paper can be accessed here:



## Supplementary Material

Additional file 1 Table 1 – data collected from OPTIMA and neuropathology records Click here for file
