# Supplementary material for: Immunohistochemical study of N-epsilon-carboxymethyl lysine (CML) in human brain: relation to vascular dementia
Source: BMC Neurol. 2007 Oct 16;7:35. doi: 10.1186/1471-2377-7-35 (PMC2100062; doi:10.1186/1471-2377-7-35)
Supplement: Additional file 1 — Table 1 – data collected from OPTIMA and neuropathology records [file 1471-2377-7-35-S1.doc]

**Table 1 – data collected from OPTIMA and neuropathology records**

| **Case**  **No** | **Age**  **(years)** | **Sex** | **CAMCOG†**  **score** | **MMSE**  **score** | **Clinical**  **dementia**  **status*** | **NIDDM?**  **Y/N** | **Hypertensive?**  **Y/N** | **PM delay**  **(hrs)** | **Cause**  **of**  **death** | **CAA**  **Y/N** | **Macro infarcts**  **Y/N** | **Lacunes**  **Y/N** | **Micro**  **Infarcts**  **Y/N** | **Small vessel subcortical disease Y/N** |
| --- | --- | --- | --- | --- | --- | --- | --- | --- | --- | --- | --- | --- | --- | --- |
| 1 | 64 | M | 64 | 19 | D | Y | Y | 144 | pneumonia | N | N | Y | N | Y |
| 2 | 83 | M | 34 | 11 | D | N | Y | 24 | pneumonia | N | Y | N | N | Y |
| 3 | 83 | M | 0 | 0 | D | N | N | 33 | pneumonia | N | Y | Y | N | Y |
| 4 | 64 | M | 98 | 26 | U | Y | Y | 23 | myocardial  infarction | N | Y | Y | Y | Y |
| 5 | 86 | F | 13 | 14 | D | N | Y | 41 | pneumonia | N | N | N | Y | Y |
| 6 | 73 | F | 13 | 3 | D | N | Y | 131 | pneumonia | N | N | Y | Y | Y |
| 7 | 83 | F | 81 | 22 | D | N | N | 72 | pneumonia | Y | N | N | Y | Y |
| 8 | 77 | M | 50 | 12 | D | N | Y | 33 | pneumonia | N | N | Y | Y | Y |
| 9 | 92 | F | 72 | 21 | D | N | N | 72 | pulmonary  embolism | N | N | Y | N | Y |
| 10 | 77 | F | 0 | 0 | D | N | Y | 77 | cardiac  arrest | N | N | N | Y | Y |
| 11 | 65 | M | 95 | 29 | U | Y | Y | 47 | pneumonia | N | N | Y | Y | Y |
| 12 | 76 | F | 102 | 29 | U | N | Y | 74 | myocardial  infarction | Y | Y | Y | N | Y |
| 13 | 77 | M | 89 | 25 | U | N | Y | 24 | pneumonia | N | Y | Y | N | Y |
| 14 | 80 | M | 102 | 29 | U | N | N | 63 | cancer | N | N | N | Y | Y |
| 15 | 77 | M | 98 | 30 | U | N | Y | 30 | Intracerebral  Haemorrhage**^**^** | Y | N | N | Y | Y |
| 16 | 76 | M | N/A | N/A | U | N | Y | 23 | cerebrovascular  accident | Y | N | N | Y | Y |
| 17 | 78 | M | 82 | 22 | U | Y | Y | 141 | pneumonia | Y | N | N | Y | Y |
| 18 | 89 | F | 52 | 13 | D | N | N | 72 | pneumonia | Y | N | N | Y | Y |
| 19 | 86 | F | 24 | 7 | D | N | Y | 40 | pneumonia | N | Y |  | Y | Y |
| 20 | 71 | F | 51 | 13 | D | N | N | 55 | pneumonia | N | Y | N | Y | Y |
| 21 | 70 | M | 95 | 27 | D | Y | N | 48 |  | N | Y | N | Y | Y |
| 22 | 92 | F | 67 | 16 | D | N | N | 85 |  | N | N | N | Y | Y |
| 23 | 88 | F | 98 | 30 | U | N | Y | 120 | pneumonia | Y | N | N | N | Y |
| 24 | 91 | M | 28 | 10 | D | N | N | 25 | myocardial  infarction | N | N | Y | Y | Y |
| 25 | 77 | F | 91 | 21 | U | N | N | 48 | myocardial  infarction | N | N | Y | Y | Y |

** Dementia status: D = dementia; U = non-demented; N/A = not available; Macro = macroscopic; micro = microscopic; CAA = congophilic amyloid angiopathy;*

*† Maximum 107; N/A=not available; NIDDM = Non insulin-dependent diabetes mellitus; **Terminal intracerebral haemorrhage in opposite hemisphere to that examined histologically*
